# A combination of burn wound injury and *Pseudomonas* infection elicits unique gene expression that enhances bacterial pathogenicity

**DOI:** 10.1128/mbio.02454-23

**Published:** 2023-11-06

**Authors:** Adrienne R. Kambouris, Jerod A. Brammer, Holly Roussey, Chixiang Chen, Alan S. Cross

**Affiliations:** 1 Center for Vaccine Development and Global Health, University of Maryland School of Medicine, Baltimore, Maryland, USA; 2 US Army Institute of Surgical Research, Joint Base San Antonio Fort Sam Houston, Houston, Texas, USA; 3 Institute for Genome Sciences, University of Maryland School of Medicine, Baltimore, Maryland, USA; 4 Division of Biostatistics and Bioinformatics, Department of Epidemiology and Public Health, University of Maryland School of Medicine, Baltimore, Maryland, USA; University of Georgia, Athens, Georgia, USA

**Keywords:** burn injury, Nanostring, *Pseudomonas aeruginosa*, innate immunity, gene expression

## Abstract

Burns are a leading cause of morbidity and mortality worldwide with the most common cause of death resulting from sepsis, often from *Pseudomonas aeruginosa*. We previously reported that a non-lethal flame burn induced an altered host immune response. Using this model, gene expression in both the murine host and *P. aeruginosa* was measured using a NanoString custom probe panel. We observed differing patterns of gene expression in both host and *P. aeruginosa* in the skin, blood, liver, and spleen of mice that were burned and/or infected, compared to mice that were neither burned nor infected (i.e., Sham). In mice that were both burned and infected (B/I), we observed changes in gene expression in both the host and *P. aeruginosa* that were distinct from all other treatment conditions. These data suggest that the combination of the burned state and superimposed infection affects both host and pathogen gene expression to increase infection propensity. Gene transcription significantly changed from 6 to 24 h post-B/I in each tissue. Finally, inhibiting IL-10 signaling or co-administering arginine at the time of *P. aeruginosa* infection prolonged or restored survival in an otherwise 100% fatal burn and infection model. These findings suggest that disease states such as burns may differentially alter innate immune response gene expression in both a host- and pathogen-specific manner.

**IMPORTANCE:**

The interaction between an underlying disease process and a specific pathogen may lead to the unique expression of genes that affect bacterial pathogenesis. These genes may not be observed during infection in the absence of, or with a different underlying process or infection during the underlying process with a different pathogen. To test this hypothesis, we used Nanostring technology to compare gene transcription in a murine-burned wound infected with *P. aeruginosa*. The Nanostring probeset allowed the simultaneous direct comparison of immune response gene expression in both multiple host tissues and *P. aeruginosa* in conditions of burn alone, infection alone, and burn with infection. While RNA-Seq is used to discover novel transcripts, NanoString could be a technique to monitor specific changes in transcriptomes between samples and bypass the additional adjustments for multispecies sample processing or the need for the additional steps of alignment and assembly required for RNASeq. Using Nanostring, we identified arginine and IL-10 as important contributors to the lethal outcome of burned mice infected with *P. aeruginosa*. While other examples of altered gene transcription are in the literature, our study suggests that a more systematic comparison of gene expression in various underlying diseases during infection with specific bacterial pathogens may lead to the identification of unique host-pathogen interactions and result in more precise therapeutic interventions.

## INTRODUCTION

Burns are a leading cause of injury and mortality worldwide. Globally, in 2017, there were almost 9 million new burn injuries due to flame, heat, or hot substances with ~121,000 reported deaths (1). A study investigating burn patient outcomes identified flames as the second most common burn source, occurring in 44% of all patients surveyed (2). Another found flame burns to be the cause of 58%–66% of all burns surveyed (3). While these patients enter treatment for burn injury, patients who succumb die from complications of infection such as shock and organ failure (4). The most common bacterial pathogens that burn patients encounter include *Staphylococcus aureus*, *Pseudomonas aeruginosa*, and *Acinetobacter baumannii* (5, 6). *P. aeruginosa* is a ubiquitous, opportunistic pathogen that causes severe infections in certain patients, such as those with cystic fibrosis, oncology patients, or those with burns (7).

Patients become infected with an opportunistic pathogen due to an impaired or failed immune response. Many models of burn injury use different burn modalities (e.g., scald, contact, chemical) or a model that results in mortality without infection. Under these conditions, it may be difficult to evaluate the effect of the burn itself on the immune response. We have used a 10% total burn surface area (TBSA) non-lethal flame burn to study the immune response to burns and subsequent susceptibility to infection. In our previous study (8), when burned mice were infected with 10^6^ CFU of *P. aeruginosa*, 100% of the mice succumbed between 24 and 36 h post-bur

In this study, we have utilized NanoString technology to simultaneously investigate gene transcription in mice and *P. aeruginosa* post-burn. This technology allows for custom panels of up to 800 gene probes that can be designed. Our panel consisted of a more manageable number including relevant host and pathogen genes (Tables S1 through 3), selected in collaboration with experts in immunology and microbiology, which allowed us to directly observe gene changes concurrently in both *Mus musculus* and *P. aeruginosa* in each sample. We chose NanoString to investigate host immune gene transcription dynamics while observing growth, virulence, and quorum sensing genes in *P. aeruginosa*. It also allows for direct comparison between samples, measuring changes in gene transcripts from a prescribed list. Finally, it is sensitive enough to measure the *P. aeruginosa* genes, which would be relatively fewer in count than *M. musculus*. Samples were collected from the blood, liver, spleen, and skin at the burn site because we previously determined that these are sites of dissemination for *P. aeruginosa* at approximately 12 h post-burn (8).

Using this probeset panel, we compared the transcriptional responses of Sham mice (mice that were clipped, received anesthesia and rehydration, but were not burned) to those of mice that were burned only (Burn) and mice that were infected only (Infection) as reference conditions. We used these conditions to determine whether mice that were both burned and infected (B/I) had unique gene expression in response to this combined treatment. We sampled tissues at multiple timepoints post-B/I to determine whether and how gene transcription changed over time in both the host and pathogen. Significantly, unique changes in gene expression of B/I mice were identified and subsequent experiments were designed to assess the efficacy of interventions that targeted these gene products.

## MATERIALS AND METHODS

### Bacterial preparation

The *P. aeruginosa* M2 isolate (IATS O5) was kindly provided by Dr. Alan Holder, formerly of the Shriners Burn Institute at the University of Cincinnati. An ice chip from *P. aeruginosa* glycerol stock was streaked for single colony isolation on tryptic soy agar (TSA) (Sigma-Aldrich, St. Louis, MO, USA) plates and incubated at 37°C for 18 h. A single colony was transferred to 3.0 mL of Hy-Soy broth, containing 0.5% sodium chloride (American Bio, Canton, MA, USA), 0.5% HY-Yeast (Kerry Bio-Science, Norwich, NY, USA), and 0.25% animal-free soytone (Teknova, Hollister, CA, USA) and grown to stationary phase at 37°C in a shaking incubator. Two hundred forty microliters of overnight inoculum was added to 12 mL of Hy-Soy broth and grown at 37°C in a shaking incubator until log phase, OD_600_ of 0.2–0.3. The bacteria were pelleted, washed twice with sterile phosphate-buffered saline (PBS), and resuspended in PBS to the desired concentration.

### Burn and infection procedure

With the approval of the University of Maryland, Baltimore IACUC, the burn procedure was performed using the Stieritz and Holder method (9) as we previously described (8, 10) Briefly, female Crl:CD1 mice (Charles River Laboratories, MA) between 8- and 10-week old had their dorsal hair clipped 24 h before the burn procedure. The following day, mice were administered 5% isoflurane for 7 min and a toe pinch was performed before each burn to ensure that the mice were successfully anesthetized. Mice were placed on their bellies in a chemical fume hood and a flame-resistant polymer card circumscribing roughly 10% TBSA (2.5 cm × 4.0 cm) was pressed down on the clipped area of the back. Five hundred microliters of 100% ethanol was deposited onto the exposed area with a glass dropper. The ethanol was ignited using a lighter and a timer was used to ensure the burn lasted exactly 10 s. The flame was extinguished by breath and immediately post-burn, mice were provided 500 µL of Ringer’s solution i.p. for fluid resuscitation. Mice were placed in their original cages for anesthesia recovery. Sham mice received the same treatment as experimental mice (i.e*.,* clip/anesthesia) except for burn and infection. To compare the responses of burned to non-burned mice to a similar bacterial challenge, mice were infected with 100 µL of 1 × 10^6^ CFU/mL (one log less than LD_50_ for non-burned mice) of *P. aeruginosa* subcutaneously at the burn site directly after the burn.

### NanoString tissue sample collection

Mice were placed under 5% isoflurane anesthesia for 7 min. Sedation was assessed by toe pinch. Mice were placed on their backs and their thoraces were sterilized with 70% ethanol. Blood samples were collected via cardiac puncture with a 1 mL syringe and 25 g needle. Whole blood was collected and stored in ethylenediaminetetraacetic acid tubes. Secondary euthanasia was completed by cervical dislocation. Post-euthanasia, a horizontal incision was completed on the dorsal aspect and the skin was resected at the burn margins. The same area was collected from sham mice. A vertical incision was then made in the abdominal cavity and the liver and spleen were resected. Each sample was stored in 3 mL of RNA*later* (Invitrogen, Thermo Fisher, MA, USA) and stored at −80°C until downstream assays were performed. The number of mice in each group at each time point is indicated in Tables S4 and S5.

### RNA isolation

RNA isolation was completed using a Trizol reagent (Invitrogen, Thermo Fisher, MA, USA), following the manufacturer’s protocol. Tissue samples were sliced using surgical scissors and weighed to be between 50 and 100 mg, then homogenized through vibration. After RNA was isolated, the samples were processed using the Monarch RNA Clean Up Kit (New England BioLabs, Ipswich, MA, USA). RNA was quantified using ThermoFisher NanoDrop Lite.

### NanoString analysis

NanoString allows for the selection of a custom panel of genes to be interrogated. Using both target and reporter probes, NanoString captures transcripts as small as 100 bp. Analysis was performed using the nCounter XT CodeSet Gene Expression Assay (NanoString Technologies) on all samples, reading the unique barcode fluorophores assigned to each gene. The samples were interrogated using a custom panel of 67 *M*. *musculus* and 32 *P*. *aeruginosa* probes (Tables S1 through 3). *Gapdh*, *Polr1b*, *Rpl19*, and *Tbp* were used as housekeeping genes for the host, whereas *oprL* and *algD* were pathogen housekeeping genes and were incorporated into the NanoString code set. The RNA was prepared and processed according to the manufacturer’s protocols. RNA (200 ng) was loaded into the codeset and hybridized for 18–20 h.

### NanoString data analysis

Analysis of raw mRNA data was completed using the NanoString Technologies nSolver analysis software version 4.0. The geometric means of eight proprietary negative control genes were used for background subtraction. The geometric means of six positive controls (minimum threshold of 0.3 and a maximum threshold of 3), along with the geometric means of the housekeeping genes (minimum threshold of 0.1 and a maximum threshold of 10) (11–13), were used to normalize the counts of each sample in accordance with the manufacturer’s protocol. The gene counts were transformed using Log_2_, and the geometric means were subtracted to calculate ratios of change between samples, then analyzed using two-way ANOVA and Tukey’s multiple comparison test. Log_10_ of *P*-values and ratios were calculated using Excel (Microsoft Corporation, WA, USA). The threshold of significance was *P* = 0.05. Data were visualized in Prism 9. Lists for Table 1 were made using Venny 2.1.0 (14).

**TABLE 1:** Shared significant gene changes in each condition reference to sham^*a*^

	(a) Present in infection only	(b) Present in burn only	(c) Present in B/I 12 h only	(d) Present in both burn only and infection only	(e) Present in both burn only and B/I 12 h	(f) Present in both infection only and B/I 12 h	(g) Present in burn, Infection, and B/I 12 h
Blood	*Il1b, Traf3,* *Myd88, Cd44, **Fcgrt***		***Tbp*** *, **Traf5, Cd40***		*Cxcr2*	*Tlr4, Chil3*	*Tlr2*
Liver		***Cxcl10***	*Tlr2, Nos2, Myd88, Il6, Ccl2, **Traf4** , Irak3, **Ddx58** , **Traf5***		*Irak2(1), Cxcl1*	***Il18***	***Abl1***
Spleen	***Traf6,Nod2, Cxcr5***	***Il6, Irak3, C8a***	*Cxcl1, **Il4,*** ***Irak2, Rag2, Irak1***	***Fcgrt***		***Abl1***	
**Skin**	*Il10, Rag2*	*Il4, Fcer1a, Myd88, Ccl2, Cd44, Polr1b, **Traf4** , Tlr2, Nos2, Cxcr5, Rag1, Cxcl10, Fasl*	***Retnla ****,* ***Irak2*** *, **C3** , **C1qa** , **Pparg** , **Il18** ,* ***Fcer1g*** *, **Traf3** , **Fcgr2b** , **Pecam1** , **Ddx58** , **Fcgr3** , oprL, **Gapdh** ,* **Tgfb1***,* ***Irak1*** *, **Traf2** ,* ***Tlr4*** *, **Casp8***		*Chil3, Cxcr2, Il6, Ptgs2, Tnf,* ***Abl1*** *, **Fcgrt** , Cxcl1, **Igf2r***	***Traf5***	*Fcgr4, Il1b*

^*a*^ Significant changes in gene expression from each tissue were analyzed using venny 2.1.0. Genes that were significantly expressed in relation to sham mice were input into the software with the groupings of burn, Infection, or B/I 12 h. The software then searched for genes that were shared among the groups. Columns are arranged to show genes that were similar within the groups (e.g., *Fcgrt* was significantly downregulated in both the Burn alone [column b] and Infection alone [column a] [but not in the B/I condition—column c] conditions in the spleen). Downregulated genes are in bold; the only *P. aeruginosa* gene is underlined in the skin—column c. Each tissue had gene transcription changes in the B/I condition that were not observed in Burn or Infection conditions. In each tissue, there were also more genes significantly impacted solely by the B/I condition that were not affected by burn alone (b) or Infection alone (a).

### IL-10 neutralization

Mice were administered one dose of either 500 µg/mL of monoclonal rat anti-mouse IL-10 neutralizing antibody (JES052A5, Invitrogen, ThermoFisher, MA, USA) or mouse IgG Isotype Control (10,400C, Invitrogen, MA, USA) in 100 µL volume i.p. 12 h after burn and infection. This timepoint was selected based on previously published measurements of IL-10 in the circulation (8), the significant expression of *Il10* in the spleen and liver at 12 h post-burn and infection, and the observed onset of clinical symptoms. Mice were monitored for survival. Significance was determined using the Log Rank Mantel-Cox test.

### Arginine supplementation

Immediately following the burn, mice were infected with 100 µL of 10^6^ CFU/mL of *P. aeruginosa* and 200 µL of 0.125 g/mL arginine (Life Technologies, ThermoFisher, MA, USA) administered subcutaneously at the burn site, superior to the burn site at the scruff of the neck, or systemically *via* i.p. injection. Serine (Life Technologies, ThermoFisher, MA USA) was used as a negative control. At the burn site, arginine (or serine) and *P. aeruginosa* were mixed immediately prior to the burn and administered simultaneously. In some experiments, arginine was administered i.p. 12 h post-burn. These doses and inoculation concentrations are consistent with reported previous work (15). Significance was determined using the Log Rank Mantel-Cox test.

To measure bacterial dissemination, blood, skin, liver, and spleen were harvested at 24 h after each treatment. Samples (0.25 g or 100 µL) were placed in 1 mL of PBS, homogenized, and then serially diluted. The dilutions were plated on TSA agar plates, incubated at 30°C overnight, then counted and log-transformed. Significance was determined using a two-way ANOVA REML mixed model with *P*-values corrected for multiple comparisons (16).

### Motility assay

*P. aeruginosa* was grown as previously described without shaking. Solutions of arginine and serine diluted in control sera were prepared at the indicated doses. An amount of 34 µL of the solutions or sera containing FlaB antibodies was placed in each well, then 1 mL of tryptic soy agar was added. The immune and non-immune sera were generated as previously described (17). Once solidified, a sharp tip was used to inoculate each well with *P. aeruginosa*. Plates were grown overnight at 30°C, images were taken, and diameters of growth were measured with ImageJ. Data were entered into GraphPad Prism, and a one-way ANOVA test with multiple-column comparison was performed.

## RESULTS

### Gene expression was modified by each treatment individually

Tissue samples from Burn mice and Infection mice were harvested at 12 h post-treatment, the time at which clinical changes were first observed in the B/I mice. In addition, the B/I mice had tissues harvested from 6 to 24 h post-treatment to assess changes over time. We utilized volcano plots to display the magnitude and significance of gene transcription when compared to a base condition (i.e*.,* 12 h B/I vs Sham was the gene expression at 12 h in mice that were burned and infected over the gene expression of Sham mice). The genes whose expression was significantly altered are shown above the dotted line at *y* = 1.3 (−log_10_ of 0.05); significance was determined using a two-way ANOVA or a mixed model test. Genes to the left of the dotted line at *x* = 0 were downregulated, while genes to the right were upregulated. *M. musculus* genes are indicated by circles and *P. aeruginosa* genes by triangles. Significantly modulated genes are indicated by open symbols.

### B/I condition impacts each tissue differentially and significantly

**Fig 1:**
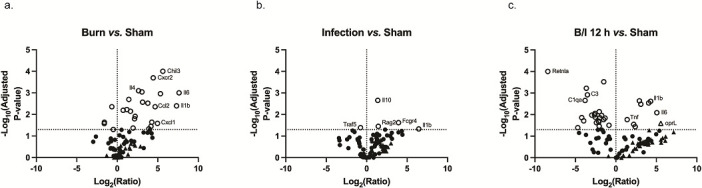
Change in gene expression in the skin in Burn alone, Infection alone, or B/I conditions compared to Sham. Mice (*n* = 5) were either subjected to a non-lethal 10% TBSA burn (Burn alone), infected s.c. with 1 × 10^6^ CFU/mL of *P. aeruginosa* strain M2 (IATS O5) in the absence of burn (Infection alone), or were both burned and infected (B/I). Sham mice had fur clipped and received anesthesia but were neither burned nor infected. All samples were collected at 12 h post-Burn or Infection and were analyzed using NanoString. Counts were transformed to the log_2_. Ratios were calculated by subtracting the geometric mean of Sham samples from the geometric mean of Burn, Infection, or B/I 12 h samples. Significance was determined using a two-way ANOVA and Tukey’s test. The −log10 of the *P*-value is plotted on the *y*-axis, with the limit of significance indicated by the dotted line at *y* = 1.3 (*P* = 0.05). Significant host genes above this line are indicated with an open circle and *P. aeruginosa* genes are an open triangle. Genes to the left of the vertical line at *x* = 0 were downregulated and genes to the right were upregulated. The closed symbols below the dotted line represent host and *P. aeruginosa* genes, respectively, in the probeset that were not significantly altered. The pattern of gene expression when compared to Sham differed in (a) Burn, (b) Infection, and (c) B/I conditions.

Compared to the same tissues in the Sham mice, the four tissues differed in response to a burn injury (Fig. 1; Fig. S1a through S3a). Overall, more genes were upregulated in the skin (Fig. 1) and blood (Fig. S1a) in response to a Burn, while the liver and spleen had more significantly downregulated genes (Fig. S2a and S3a). The skin from mice that were burned alone had a high number of significantly upregulated genes (Fig. 1a). The blood was the least affected, with increased transcription of only *Tlr2* and *Cxcr2* (Fig. S1a). In the spleen and liver, the overall pattern was downregulation of gene expression (Fig. S2a and S3a).

As was observed in the Burn condition, the Infection condition caused increased upregulation of host genes in the skin (Fig. 1b) and blood (Fig. S1b) while the liver and spleen had more downregulation (Fig. S2b and S3b). The skin and blood showed an increase in transcription of pro-inflammatory mediators and neutrophil chemokine genes in response to *P. aeruginosa* infection over the Sham condition. The liver (Fig. S2b) and spleen (Fig. S3b), however, were less affected. From these figures, we determined that both the Burn condition and the Infection condition impacted gene transcription differently.

When visualizing the B/I condition, the previously observed patterns in each of the tissues of the Burn only and Infection only conditions were not maintained. There were both significantly up- and downregulated genes at 12 h in the skin (Fig. 1c). The *P. aeruginosa* gene, *oprL*, was only significantly upregulated in the B/I condition (Fig. 1c). The tissues distal to the burn site were also differentially altered by the B/I condition. There were both significant up- and downregulation changes in the blood, spleen, and liver (Fig. S1c through S3c).

### The B/I condition affects unique genes in all tissues

The Burn only and Infection only samples were collected at 12 h post-treatment, when clinical symptoms for B/I mice first appeared, which allowed for direct comparison with samples collected at 12 h post-B/I. To compare the three conditions, genes significantly up- and downregulated in relation to Sham (e.g., *P*-value ≤ 0.05) were analyzed by the Venny 2.1.0 program. We sought to confirm whether the B/I condition impacted gene regulation differently than it did for Burn alone or Infection alone. Venny 2.1.0 analysis created groups of similarly expressed genes that are identified in Table 1. Downregulated genes are indicated in bold and upregulated genes are not in bold; host genes are in normal text and the only *P. aeruginosa* gene is underlined.

In each tissue, there were genes significantly and uniquely affected by the B/I condition (Table 1). While in some instances, the same genes were similarly transcribed in all three conditions, the overall transcription patterns appeared to differ in the three conditions and all four tissues. Specifically, in the skin, there were two genes (*Fcgr4* and *Il1b*) that were commonly upregulated under all conditions (Table 1g). Infection caused only two uniquely upregulated genes, *Il10* (IL-10) and *Rag2* (Table 1a). There were several genes that were upregulated and one downregulated in response to Burn only (Table 1b). By contrast, the B/I condition caused one uniquely upregulated and 18 uniquely downregulated genes in the skin (Table 1c). The uniquely upregulated gene in the B/I condition was a *P. aeruginosa* gene, *oprL*, which is a peptidoglycan-associated lipoprotein (Table 1c). In the blood, there were three significantly downregulated genes in the B/I condition and one gene commonly upregulated in all three conditions, *Tlr2* (Table 1g). The liver and spleen followed the same pattern, with the B/I condition changing expression in the highest number of genes, nine and five, respectively (Table 1c).

**Fig 2:**
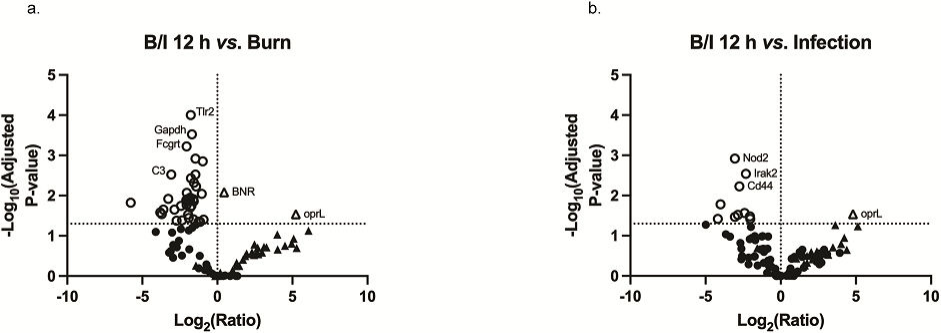
Change in gene expression in the skin in the B/I condition compared to Burn alone or Infection alone condition at 12 h. When calculating the gene change ratio, either Burn or Infection alone gene counts were used as the baseline. The figures are oriented as previously described in Fig. 1. In comparison to both reference conditions, the B/I condition resulted in a high number of downregulated host genes. In fact, the only upregulated gene in the B/I condition when compared to (**A**) Burn and (**B**) Infection alone are the *Pseudomonas* genes, *oprL*.

To determine the magnitude of gene expression in relation to the Burn alone and Infection alone conditions, we compared gene expression in the skin in the B/I condition to both the Burn alone and Infection alone conditions in volcano plots. When compared to Burn alone, there were no significantly increased host genes at 12 h B/I (Fig. 2a). There was, however, decreased expression of numerous genes in the host panel. At 12 h B/I, *P. aeruginosa* had significantly upregulated *oprL* when compared to the Burn condition (Fig. 2a). In reference to Infection, the B/I condition had downregulation of several inflammatory mediator genes. In sum, both the host and pathogen had altered gene expression in the B/I condition (Fig. 2). When using Burn or Infection conditions as the baseline, the B/I condition had uniquely and significantly impacted genes in all tissues in the host (Fig. S1 through S3d, e).

### Kinetics of gene expression changes over time in the B/I condition

**Fig 3:**
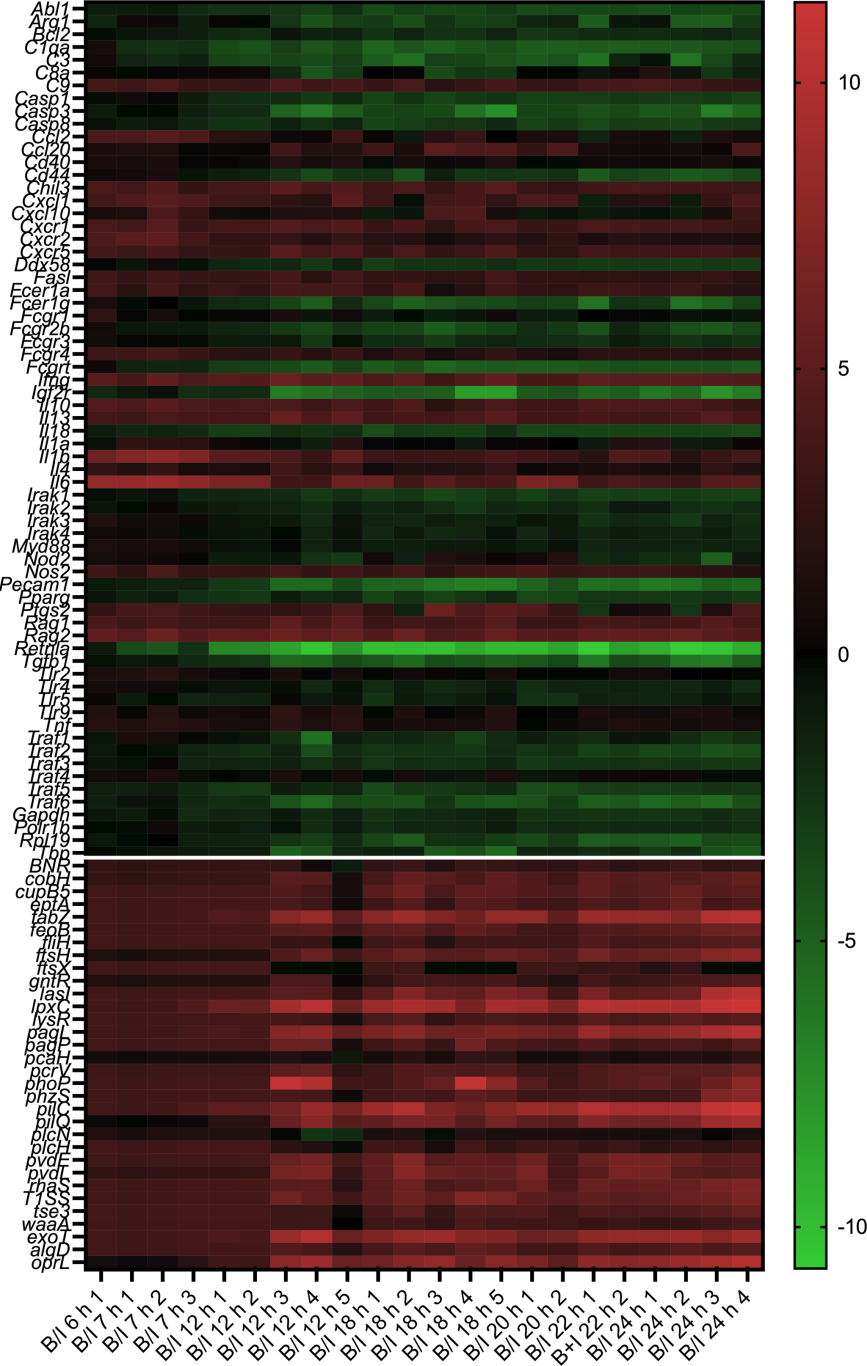
Gene ratios in the skin over time in the B/I condition. A heatmap illustrates how gene ratios changed over time. The ratio was calculated as the gene expression in B/I mice compared to the Sham condition. Each column on the heatmap is the sample collected from one mouse. The columns are organized increasing in time from left to right, starting at 6 h post-B/I and ending at 24 h. Each row is an interrogated gene in the Nanostring panel (Tables S1 through 3). Genes above the horizontal white line are from *M. musculus* and below are from *P. aeruginosa*. An increase in ratio is indicated in red and a decrease in green. The figure qualitatively suggests that after a period of relative quiescence from 6 to 12 h post-B/I, there is a clear divergence in expression patterns between the host and the pathogen: there is a global decrease in host gene expression and an increase in pathogen gene expression from 12 to 24 h.

**Fig 4:**
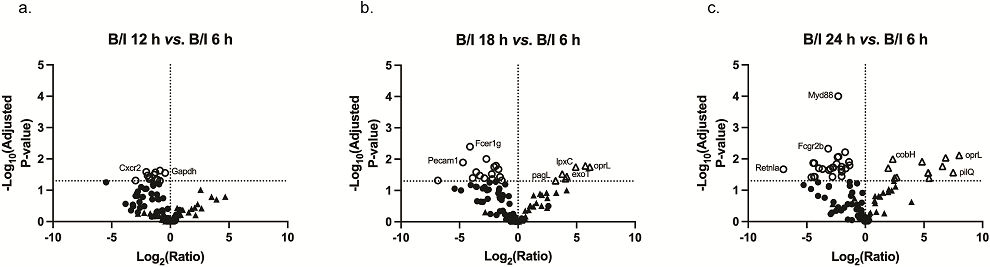
Gene expression in the skin over time in the B/I condition using gene expression at B/I 6 h as baseline. Gene ratios were calculated using the geometric mean at 6 h post-B/I subtracted from the geometric mean of gene expression at (a) 12 h, (b) 18 h, and (c) 24 h post-B/I. Statistical significance was determined using a two-way ANOVA. Figures are oriented as previously described. As the time of skin harvest increased, there were more host genes downregulated (shift to left) and more *P. aeruginosa* genes upregulated (shift to right).

In the B/I condition, the profile of gene expression in the skin changed over time, as visualized in the heatmap (Fig. 3). The heatmap expresses the ratio of log_2_ of gene expression over the geometric mean of the five Sham condition samples. Above the horizontal white line are *Mus musculus* genes, while *Pseudomonas aeruginosa* genes are depicted below. Samples were obtained over time from 6 to 24 h (left to right). Each column represents one mouse. The map shows a generalized period of quiescence from 6 to 12 h in both the host and *P. aeruginosa*. At 12 h post-B/I, when clinical symptoms first appeared, there was a predominate downregulation of genes in the host, while *P. aeruginosa* exhibited an increase in gene expression. This can be more clearly visualized with volcano plots (Fig. 4).

To determine the magnitude of gene change over time, gene count ratios and significance were calculated with the B/I 6 h time point as the baseline. *M. musculus* genes that were significantly modulated are indicated by an open circle while significantly changed *P. aeruginosa* genes are shown as open triangles. At B/I 12 h, several host genes significantly decreased in transcription (Fig. 4a). At 18 h, a pattern emerged, mirroring the observations in the heatmap (Fig. 4b). The significantly upregulated genes were solely the *P. aeruginosa* genes, including the LPS synthesis gene *lpxC* and toxin gene *exoT*; the number of host genes that were significantly downregulated increased. This trend continued through 24 h (Fig. 4c). The changes in gene transcription in the skin under the B/I condition were significantly different as time progressed, with a decrease in host gene expression simultaneously with an increase in pathogen gene expression.

### Neutralizing IL-10 prolongs survival

**Fig 5:**
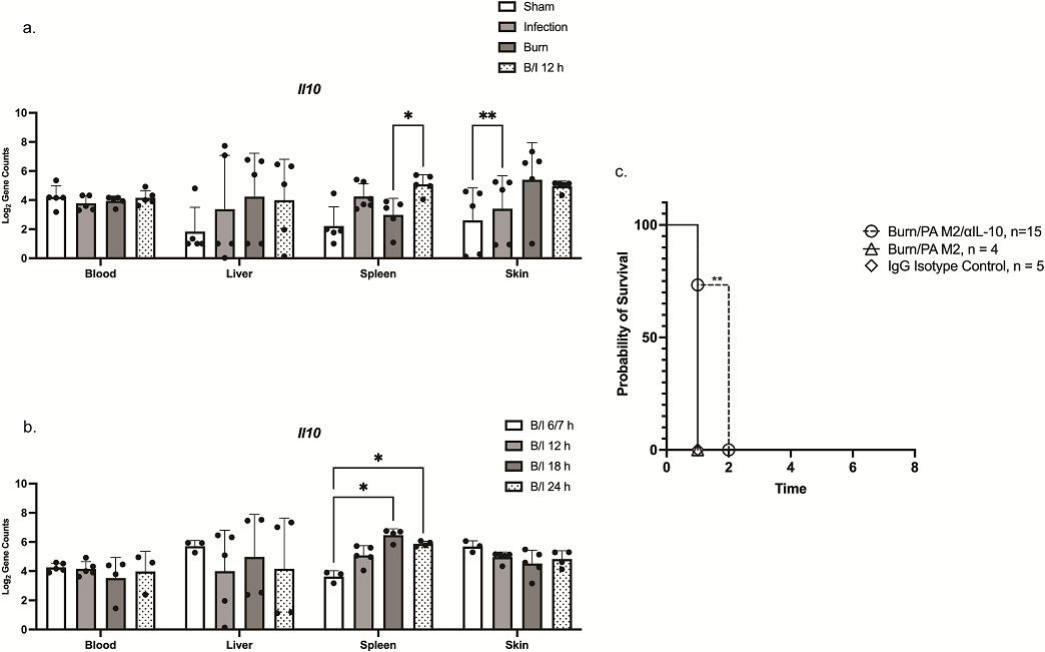
IL-10 neutralizing antibody prolonged survival. The bars represent the mean of Log_2_ of *Il10* gene transcript counts in each tissue and each dot represents an individual mouse. Error bars indicate standard deviation. Significance was determined using an REML mixed model (16). (**A**) The Infection, Burn, and B/I gene counts are at 12 h post-treatment. There is significant expression of *Il10* at in the spleen in the B/I condition compared to the Burn condition. (**B**) Gene expression in each tissue over time in the B/I condition. Transcription of *Il10* is significantly increased at 18 and 24 h post-B/I in the spleen. (**C**) Kaplan-Μeier plot of survival of mice that were administered anti-IL-10 neutralizing monoclonal antibody. Significance was measured using a Log Rank Mantel-Cox test. Antibody was administered i.p. at 12 h post-B/I; mice had prolonged survival of 1 day (Day 1: No α-IL-10 0/4 vs α-IL-10 11/15). For panels a–c: *P < 0.05, **P < 0.01.

We sought to identify host gene transcripts that were impacted by B/I and determine whether they exerted a role in the observed decrease in host defense post-burn. IL-10 is an anti-inflammatory mediator primarily produced by monocytes (18). It inhibits cytokine production in the Th1 response (18). Previous work in our laboratory showed that in this model, IL-10 protein was found circulating in the blood of mice at 12 h post-B/I, peaking at 18 h, and persisting until death (8). We also saw a transient increase in infection susceptibility, with mice succumbing between 20- and 30 h post-B/I (8). In the NanoString data from the spleen, there was a significant increase at 12 h in transcription of *Il10* in the B/I condition compared to the Burn alone condition (Fig. 5A) and *Il10* transcription significantly increased from 6 to 24 h (Fig. 5B). Importantly, there was no significant change in transcription in the blood, suggesting that the source of IL-10 protein previously observed was from distal tissues such as the skin or spleen.

Based on a significant transcription of *Il10* in the spleen, we attempted to inhibit this anti-inflammatory mediator to determine its impact on survival. Since the significant increase in transcription began in the spleen at 12 h post-B/I, the neutralizing anti-IL-10 antibodies were delivered i.p. at 12 h post-B/I. Mortality was significantly delayed in anti-IL-10-treated mice by 1 day (day 1 survival: no antibody 0/4, antibody 11/15, *P* = 0.0102, log rank Mantel-Cox) (Fig. 5C). An additional dose at 24 h post-B/I, doubling the initial dose of antibody or irrelevant isotype control monoclonal antibody did not enhance survival or further delay death (data not shown). It is likely that the sustained production of IL-10 by the tissues overwhelmed the antibody treatment.

### Site of arginine administration impacts mortality

Arginine is an amino acid utilized in the host immune response. Depending on the activating immune environment, arginine can be metabolized in two ways: either by arginase (*Arg1*) in M2 macrophages in response to a Th2 immune response or by inducible nitric oxide synthase (iNOS; *Nos2*) produced by M1 macrophages during a Th1 response (19). Arginase, whose expression is a marker for M2 macrophages (20–22), is a key component of the resolution of inflammatory response and tissue repair. If arginase predominates in this system, arginine is metabolized into precursors for collagen, which aids in the wound healing process (19). In a pro-inflammatory environment, iNOS metabolizes arginine during the production of nitric oxide, which is bactericidal. Nitric oxide inhibits arginase activity and collagen precursors inhibit iNOS (19).

Arginine also plays a role in the pathogenesis of *P. aeruginosa* post-burn. It can serve as a carbon source under anaerobic conditions and *P. aeruginosa* grown on arginine-enriched plates has decreased motility *in vitro* (15). In the same study, administering arginine s.c. at the burn site at the time of burn and infection *in vivo* increased survival (15). If arginase or iNOS expression were high, it would diminish the amount of available arginine to inhibit the motility and therefore promote dissemination of *P. aeruginosa*.

High transcription of *Arg1* would be expected to result in depletion of available arginine and promote *P. aeruginosa* dissemination; however, arginase gene expression was not significantly increased in any tissue or conditions (Fig. S4a and b). *Nos2* expression, however, was significantly increased in the liver and blood of the B/I condition at 12 h (Fig. S4c) and increased from 6 to 12 h post-B/I in the blood (Fig. S4d). The expression in the skin in the B/I condition was not significantly impacted (Fig. S4c and d). If arginine were high in the immediate burn environment, a decrease in *P. aeruginosa* dissemination would be predicted. Therefore, we hypothesized that the effect of arginine on survival in B/I mice would be due to its co-localization with *P. aeruginosa* and its effect on motility, rather than the tissue expression of *Arg1* and *Nos2*.

To test this hypothesis, 0.125 g/mL of arginine was administered immediately post-burn either s.c. or i.p. to determine whether it affected host responses. The s.c. doses were given either concurrently with *P. aeruginosa* at the burn site or superior to the burn site, and distal to the *P. aeruginosa*, at the scruff of the neck of the mouse. The i.p. dose was to evaluate the effect of systemic arginine. These sites were chosen to determine the relationship between the location of arginine and *P. aeruginosa* dissemination. The mice were then monitored for survival. All statistics in the survival plots were calculated using the log rank Mantel-Cox test.

**Fig 6:**
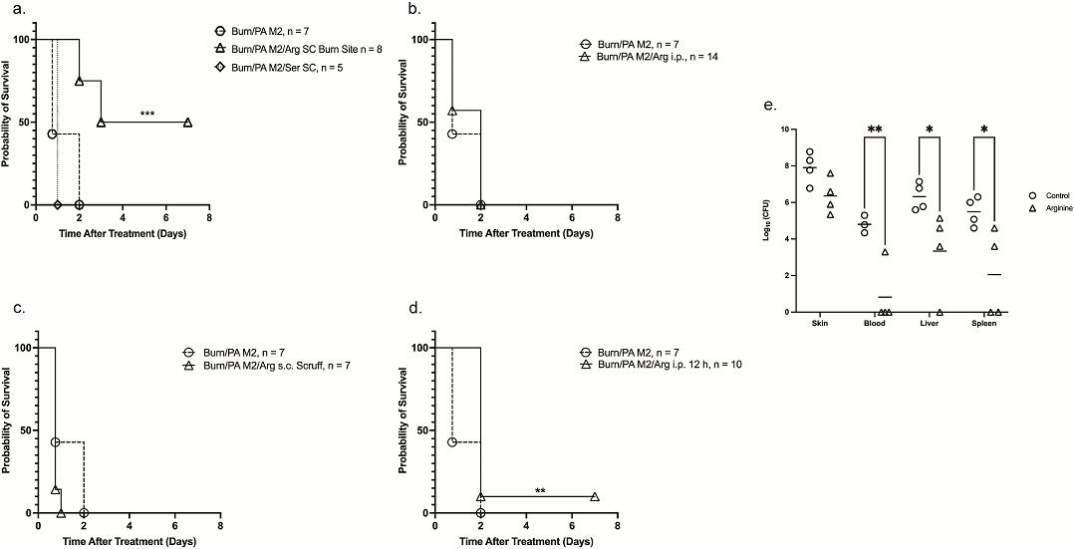
Arginine restored survival in B/I mice and decreased dissemination. Mice were administered 200 µL of 0.125 g/mL of arginine or serine immediately or 12 h post-B/I; the number of mice in each treatment is indicated in the legend. Figures represent combined data from 2 to 3 individual experiments. (a) Arginine, serine, or PBS was mixed with the *P. aeruginosa* inoculum and immediately delivered s.c. at the burn site. (b) Arginine was administered s.c. in the scruff of the neck, distal to the burn and infection site, immediately post-B/I. (c) To measure systemic effects, arginine was administered i.p. immediately post-B/I. (d) Arginine was administered i.p. 12 h post-B/I. Arginine restored survival to mice when administered s.c. immediately post-B/I and i.p. 12 h post-B/I (day 1: no arginine 3/7 vs arginine 10/10). One mouse survived for 7 days. (e) Mice were burned and either infected s.c. with *P. aeruginosa* in PBS or *P. aeruginosa* in arginine. At 24 h post-B/I, skin, liver, spleen, and blood samples were homogenized in PBS, serially diluted, and plated on TSA agar plates. After overnight incubation at 30°C, individual colonies were counted and log transformed. Significance was determined using an REML mixed model (16). Although there was an active infection in the skin of all mice, there was a significant decrease in dissemination to distal tissues in mice that received arginine. *P < 0.05, **P < 0.01.

Delivering arginine s.c., concurrently with *P. aeruginosa* infection, post-burn at the burn site resulted in 50% of mice surviving when compared to B/I alone (No Arginine 0/7 vs Arginine 4/8, *P* = 0.0013, log rank Mantel-Cox) (Fig. 6a). This appeared to be a local effect on the pathogen, as administering arginine distally in the scruff of the neck immediately following B/I caused the mice to succumb sooner (no arginine 0/7 vs arginine 0/7) (Fig. 6b). Arginine was also delivered systemically via i.p. injection immediately post-B/I, but, here, too, there was no impact on mortality (no arginine 0/7 vs arginine 0/14) (Fig. 6c). Based on previous data showing the presence of *P. aeruginosa* in the blood at 12 h post-B/I, we administered arginine i.p. at 12 h post-B/I and observed prolonged survival at day 1 in the arginine-treated mice (no arginine 3/7 vs arginine 10/10) and that one of the mice even survived to day 7, *P* = 0.0085 (Fig. 6d). Administering a similar dose of serine s.c. at the burn site at the time of infection did not impact survival (Fig. 6a).

To determine whether the delayed deaths were due to decreased *P. aeruginosa* dissemination, we repeated administering arginine s.c. at the burn site immediately post-B/I and measured bacterial burden in tissue samples (0.025 g or 100 uL) at 24 h post-treatment. While there was still active infection at the burn site in all mice at 24 h, arginine induced a significant decrease in the dissemination of the organism to organs (Fig. 6E). In some mice, there were no CFUs found in distal tissues. Administering arginine s.c. at the burn site at the time of infection decreased dissemination of *P. aeruginosa* and increased survival in 50% of mice, suggesting that supplemental arginine in the skin B/I environment decreased the dissemination of *P. aeruginosa* to distal tissues. We speculate that the arginine effect in our studies was due to its local effect on *P. aeruginosa*, inhibiting growth and impacting motility (Fig. S5), rather than acting on a host defense response.

### Mice infected with *P. aeruginosa* incubated with arginine are fully protected

We speculated that the observed delay in death in mice that were administered arginine was due to its impact on *P. aeruginosa* motility. To test this hypothesis, the same experiment as described above was repeated with the added variable of infection with *P. aeruginosa* that was incubated with the same dose of arginine for 1 h prior to the burn. The mice were then either infected with *P. aeruginosa* and given a s.c. dose of arginine (PA M2/s.c. Arg) or infected with *P. aeruginosa* from the incubation (PA M2/Arginine). This was conducted in Sham mice and mice that were burned. There were also control groups of mice that were administered *P. aeruginosa* without arginine (PA M2).

**Fig 7:**
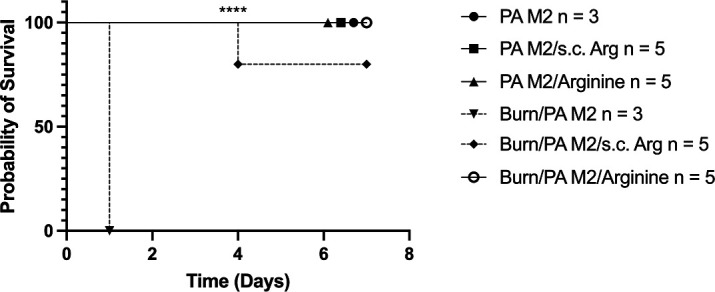
Burned mice infected with *P. aeruginosa* incubated with arginine had 100% survival. Mice (±Burn) were infected with 10^6^ CFU of *P. aeruginosa* (PA M2), infected and administered 0.125 g/mL subcutaneously (PA M2/s.c. Arg), or infected with *P. aeruginosa* which had been incubated with 0.125 g/mL of arginine for 1 h (PA M2/Arginine). The mice were monitored for survival for 7 days. All mice that had not been burned survived (closed circle, square, and triangle). Mice that were burned and infected without arginine died by 24 h (inverted triangle). Mice that received arginine s.c. at the time of B/I had prolonged survival (diamond). The burned mice infected with PA incubated with arginine (PA M2/Arginine) had 100% survival (open circle). ****P < 0.0001

As previously observed, the untreated B/I mice (inverted triangle) succumbed at 24 h post-treatment, but the B/I mice that received s.c. arginine (diamond) had a high survival rate and delayed time to death (Fig. 7). As expected, the mice that were infected but not burned had 100% survival as well (closed circle). Interestingly, the mice that were burned and infected with the arginine-incubated *P. aeruginosa* also exhibited 100% survival (open circle). These results indicate that survival in these mice was most likely due to direct interaction between *P. aeruginosa* and arginine.

## DISCUSSION

The goal of the present study was to determine whether our previously observed host/pathogen interactions were due to changes in gene transcription, in either the host or *P. aeruginosa,* which may explain the increased mortality in mice that were burned and infected. Gene transcription in the host was impacted by both burn alone and infection alone. When done concurrently, however, a burn and infection caused changes in gene transcription in each host tissue interrogated that were not seen in the other conditions. Furthermore, in burned and infected animals, there were genes that were significantly upregulated in *P. aeruginosa* that were not upregulated during an infection alone. This shows that the burned state of the skin impacted gene transcription not only in the host but also in *P. aeruginosa*. The combination of burn and *P. aeruginosa* infection significantly and uniquely impacted both the host and the pathogen at the level of transcription.

We previously hypothesized that the skin served as a reservoir for *P. aeruginosa* growth and dissemination. Our NanoString data support this hypothesis. Over time, there was a global downregulation of host genes in the skin. This could not be attributed to dead cells: while the epidermis and dermis were destroyed in our burn model, the underlying support structures remained intact (10). When B/I skin was H&E stained, there was an increase in recruited lymphocytes in comparison to Burn only skin (10). This suggests that the changes in gene transcription in the host skin were due to cells that were recruited to the burn site, such as neutrophils and monocytes. Furthermore, in the Burn alone and Infection alone conditions, there was significant upregulation of host genes at 12 h, but when the B/I condition was compared to the Burn or Infection alone conditions, expression of these same host genes was significantly decreased in expression. *P. aeruginosa* had one gene significantly expressed in reference to Sham, *oprL*, which is an outer-membrane bound lipoprotein. Significant expression of *oprL* at 12 hours post-B/I supports the hypothesis that the increase in transcripts is likely due to an increase in the total number of *P. aeruginosa* rather than an upregulation of gene expression in individual bacteria. Because of its role in peptidoglycan synthesis, the *opr* operon has been utilized as a vaccine target (23–25).

When investigating the changes in gene expression in the skin over time, we can better understand the gene expression of the pathogen. Using B/I 6 h as the baseline reference point for *Pseudomonas* gene expression, at 18 h there was a significantly increased transcription of *lpxC* and *pagL*, genes involved in altering the structure of LPS. This could result in immune evasion (26, 27). As the B/I condition progressed over time, there was a significant upregulation of pili genes *pilC* and *pilQ*, protease *ftsH*, and toxin *exoT*, a virulence factor shown to aid in the dissemination of *Pseudomonas* (28). The *P. aeruginosa* M2 strain lacked the *exoU* gene (29).

Previous literature using dual-RNASeq has shown that *P. aeruginosa* gene expression and kinetics vary based on the wound and disease type (30–37). In models of pneumonia and cystic fibrosis, *P. aeruginosa* has been found to increase iron acquisition gene expression (31, 37). In the comparison of gene expression in an acute scald burn wound and chronic surgical wound on the same animal, they found that *P. aeruginosa* had greater gene expression in genes related to cell motility and cell envelope biogenesis in the burn wound than in the chronic wound (35). Similar to our work, a study compared the transcriptome of *Pseudomonas* grown in burn wound exudate (BWE) or broth (32). They found that there were genes uniquely altered in the BWE condition, similar to our B/I findings (32). These works all show that *P. aeruginosa* and the host alter gene expression differentially based on the various underlying conditions but is difficult to compare to other published work. We suggest that the relationships could be better studied and compared using a standardized NanoString panel. Understanding these specific host and pathogen gene changes, if proven to be unique, could result in precision treatment for specific disease/infection states.

RNA was isolated from whole blood. The assumption was that any gene changes observed in the blood were due to circulating leukocytes since red blood cells and platelets do not have nuclei, although we cannot ignore the possibility that these transcripts can be due to preformed mRNA or parenchymal cells in circulation because of the burn. Changes in gene transcription in the blood were minimal; there was a significant decrease in Il1b (IL-1β) transcription when comparing the B/I to the Infection condition at 12 h. This finding would support the dissemination of *P. aeruginosa*. High circulating IL-1β levels have been positively correlated with infectious disease severity (38–40). Also, IL-1β is important in protection against infection (41). The fact that *Il1b* transcription decreased in the B/I condition and that circulating concentrations of IL-1β protein did not significantly increase post-B/I (8), indicates an inadequate immune response which may predispose the mice to bacterial dissemination and lethality. This can also account for, in part, the decreased LD_50_ of *P. aeruginosa* infection post-burn.

When investigating the transiently increased infection susceptibility post-burn, we looked for significant gene expression that would contribute to an anti-inflammatory response. In previous work, IL-10 was elevated in the blood at 18 h post-burn and infection and remained elevated until the mice succumbed (8). IL-10 is an autoregulatory cytokine released primarily by monocytes (18). It inhibits the synthesis of cytokines in the Th1 response through activating STAT3, which is the transcription factor for anti-inflammatory cytokines (18, 42). It has been found in the circulation of trauma patients, in correlation with injury severity (43). It has also been positively associated with post-traumatic infection susceptibility and mortality (43–46). While transcription in the liver and spleen was not as impacted by the B/I condition as in the skin, *Il10* (IL-10) was transcribed significantly in both distal tissues. Using anti-IL-10 neutralizing antibodies, we found that neutralizing IL-10 post-B/I increased survival when administered 12 h post-B/I, which was the observed clinical onset of symptoms. Giving an additional dose at 24 h, or doubling the initial IL-10 dose, did not further increase survival or extend mean time to death, possibly due to the continued transcription of *Il10* by the spleen. Higher and earlier doses of anti-IL-10 may be able to further improve survival in our burn model.

Another relationship we investigated was the effect of arginine on infection susceptibility post-burn. In the host, arginase is an enzyme involved in the urea cycle in the liver and arginine metabolism in immune cells (47). Recruited macrophages alter the metabolism of arginine based on the surrounding cytokines (19, 22, 47). Pro-inflammatory mediators cause an upregulation of iNOS, resulting in nitric oxide production, which is bactericidal. In anti-inflammatory environments, arginase is upregulated, which uses arginine to form precursors to collagen, thereby promoting wound healing (47). The pathogen, *P. aeruginosa* can utilize arginine as a source of ATP in anaerobic conditions through the arginine deaminase pathway (48). Arginine inhibited swarming motility in a dose-dependent manner, and did so more significantly than other amino acids at the same dose (49). It has also been associated with increased biofilm formation (49–51). *P. aeruginosa* with an induced loss of swarming motility was found to have decreased dissemination to distal tissues (52–54).

Previous work in a non-lethal scald burn and infection model implicated arginine as a factor in *P. aeruginosa* pathogenesis post-burn (15). It was shown to limit the motility of *P. aeruginosa in vitro* when grown on arginine-enriched agar (15). Myeloid-derived suppressor cells (MDSCs) were hypothesized as the source of arginase activity, which would decrease the arginine availability and aid in *P. aeruginosa* dissemination.

In our studies, administering arginine immediately at the burn site or 12 h post-B/I i.p. significantly prolonged and increased survival. This effect was location dependent and time dependent, as administration of arginine in the scruff (superior to the burn site) or immediately after burn and infection i.p. did not improve survival. The arginine dose used also decreased the dissemination of *P. aeruginosa*, likely due to an inhibition of growth and motility. Furthermore, the administration of *P. aeruginosa* incubated for 1 h with arginine resulted in 100% survival of the mice. These results suggest that *P. aeruginosa* and arginine must be co-located physically and temporally for the effect on motility and dissemination to be observed. The increased survival in our study is more likely due to the arginine/*P. aeruginosa* interaction rather than being attributed to the effect of arginine on host defenses. And while MDSCs express arginase (55–57), and have been shown to be recruited to a burn site by day 3 post-burn (58), the interaction between arginine and *P. aeruginosa* appeared to be more acute.

Our study had several limitations. We wanted to investigate specific pathways known to be affected by burn and infection alone, such as those involved with innate immunity, cell death, inflammatory mediators, and bacterial virulence factors (59–61). While the use of NanoString allowed us to evaluate a custom panel of genes quickly and with high sensitivity, it introduced selection bias. RNA-Seq would remove selection bias by interrogating all transcripts over the threshold of sensitivity; however, NanoString allowed us to begin analyzing data without the need for steps such as alignment and assembly, which RNASeq requires. Multi-species RNASeq requires additional adjustments in sample processing (62).

Since previous work has shown dissemination of *P. aeruginosa* in the blood, liver, and spleen post-burn (8), we chose these sites for sample collection. In patients with burn wounds, positive *P. aeruginosa* cultures were found in skin, urine, sputum, and blood (63). The most common cause of death post-burn is sepsis with multi-organ failure (3, 4, 64, 65); patients who died from sepsis post-burn had symptoms of kidney and liver failure (66). Consequently, we may not have a complete picture of host/pathogen interactions by not sampling additional sites (e.g., kidneys).

While fold change can be calculated using housekeeping genes as the baseline, we observed that the housekeeping genes that we chose, both for the host and the pathogen, were not consistently expressed, especially in the skin. Furthermore, external variables such as carbon sources and antibiotics can alter the expression of common housekeeping genes in *P. aeruginosa* (67, 68). We found that the variations in gene expression for our selected “housekeeping” genes were insufficient to serve as a basis for comparison. For these reasons, we chose to use the individual genes at the indicated baseline conditions (Sham, Burn, etc.) as the baseline for ratio calculations.

We found that gene transcription was impacted in a significantly different manner when mice were concurrently burned and infected with *P. aeruginosa* than if they were burned alone or infected alone. This was observed in both the host and the pathogen. If mice were dosed with α-IL-10 or arginine at specific sites and timepoints, increased survival was observed. Future directions should include further optimization of the α-IL-10 doses, determining whether other anti-inflammatory mediators impact survival, and investigating whether additional gene pathways are impacted by burn and infection.

Importantly, our work suggests that underlying disease states in combination with infection can alter gene transcription both in the pathogen and host, which may affect outcomes. It may be that different disease states such as trauma or diabetes mellitus, while altering host gene expression in a disease-specific manner, like burn injury, also may impact the expression of genes in a pathogen-specific manner. The host environment may significantly alter components of the bacterial cell wall as may have occurred in our studies with an increase in *oprL* and *lpxC* which affect cell integrity and lipid A synthesis, respectively. Furthermore, pathogen gene expression in one disease state may differ from sets of genes expressed in other disease states. If true, this introduces an entirely new complexity in studying host-pathogen interactions. While many papers similarly examine host and bacterial gene expression under various conditions, few systematically compare specific gene expression under different disease states. Such a study could identify novel disease-associated host immune pathways that may be associated with specific pathogens. NanoString provides the opportunity to make these standardized observations. Such insights could lead to unique therapeutic interventions.

## SUPPLEMENTAL MATERIAL

Figure S1 mbio.02454-23-s0001.tif Change in gene expression in the blood in Burn alone, Infection alone, or B/I conditions compared to Sham. Experiment completed and figures prepared as described in Fig. 1. There were minimal gene changes in the blood, but each condition affected the blood differently (a-c). The B/I condition was more similar to Burn alone and Infection alone, with minimal gene changes in the B/I conditions (d, e). There was no discernible pattern of expression over time in the blood; however, by 24 h, a *P. aeruginosa* gene, *gntR*, a transcriptional regulator involved in glucose metabolism, was significantly upregulated (f-h).

Figure S2 mbio.02454-23-s0002.tif Change in gene expression in the liver in Burn alone, Infection alone, or B/I conditions compared to Sham. Experiment completed and figures prepared as described in Fig. 1. There were more genes affected in the B/I condition in both up- and downregulated genes compared to either Burn alone or Infection alone (a-c). In reference to both Burn alone and Infection alone, the B/I condition caused more upregulation of pro-inflammatory mediators (d, e). Host gene expression, but not *P. aeruginosa*, increased in ratio over time but not in the number of significantly expressed genes (f-h).

Figure S3 mbio.02454-23-s0003.tif Change in gene expression in the spleen in Burn alone, Infection alone, or B/I conditions compared to Sham. Experiment completed and figures prepared as described in Fig. 1. There is only one upregulated gene in any of the conditions, *Cxcl1* in the B/I condition. All other conditions caused a downregulation of genes (a-c). There was an increase of *Cxcl1* over both the Burn alone and Infection alone, however there was a downregulation of *oprL* compared to Infection alone. *Il10* was increased in expression when compared to the Burn alone condition (d, e). Upregulated host genes increased in number over time, but there were no pattern in ratio changes. *Il10* expression was upregulated and maintained through 24 h post-B/I (f-h).

Figure S4 mbio.02454-23-s0004.tif Changes in gene expression of *Arg1* and *Nos2* in each condition and over time in the B/I condition. The bars represent the mean of the log_2_ of *Arg1* and Nos2 gene transcript counts in each tissue and each dot represents an individual mouse. Error bars indicate standard deviation. Significance was determined using an REML mixed model with multiple comparisons (16). There was no significant increased expression of *Arg1* in any treatment condition (a) or any significant changes in expression over time in the B/I condition (b) in any tissues. *Nos2* was significantly expressed in the liver and blood in the B/I condition (c) but not in the skin. Its expression increased from 6-12 h in the blood (d).

Figure S5 mbio.02454-23-s0005.tif Arginine inhibits motility and growth of *P. aeruginosa*. (a) *P. aeruginosa* was grown in tryptic soy agar (TSA) wells supplemented with rabbit sera with FlaB antibodies (FlaB), or increasing doses (100-750 mM) of arginine or serine diluted in normal rabbit serum. *P. aeruginosa* had high motility at 250 mM which decreased in a dose-dependent manner at 750 mM. Serine did not have this effect. (b) *P. aeruginosa* grown in the bacterial culture medium, Hy-Soy broth, supplemented with 100 mM or 500 mM of arginine. Spectrophotometer readings were taken at OD_600_ to measure *Pseudomonas* growth. The lower dose of arginine allowed growth at a rate comparable to that in Hy-Soy broth; however, the higher dose inhibited growth. One-way ANOVA and Tukey's test with multiple comparisons. *, *P*, < 0.05; **, *P*, < 0.01; ***, *P*, < 0.001.

Table S1 mbio.02454-23-s0006.pdf *M. musculus* genes included in NanoString panel.

Table S2 mbio.02454-23-s0007.pdf *P. aeruginosa* genes included in NanoString panel.

Table S3 mbio.02454-23-s0008.pdf *M. musculus* and *P. aeruginosa* housekeeping genes included in NanoString panel. *P. aeruginosa* genes are underlined.

Table S4 mbio.02454-23-s0009.pdf Number of mice sampled in each reference condition and tissue.

Table S5 mbio.02454-23-s0010.pdf Number of mice sampled in the B/I condition in each tissue per timepoint.

ASM does not own the copyrights to Supplemental Material that may be linked to, or accessed through, an article. The authors have granted ASM a non-exclusive, world-wide license to publish the Supplemental Material files. Please contact the corresponding author directly for reuse.
