# Supplementary figures and images for: A combination of burn wound injury and Pseudomonas infection elicits unique gene expression that enhances bacterial pathogenicity
Source: mBio. 2023 Nov 6;14(6):e02454-23. doi: 10.1128/mbio.02454-23 (PMC10746159; doi:10.1128/mbio.02454-23)

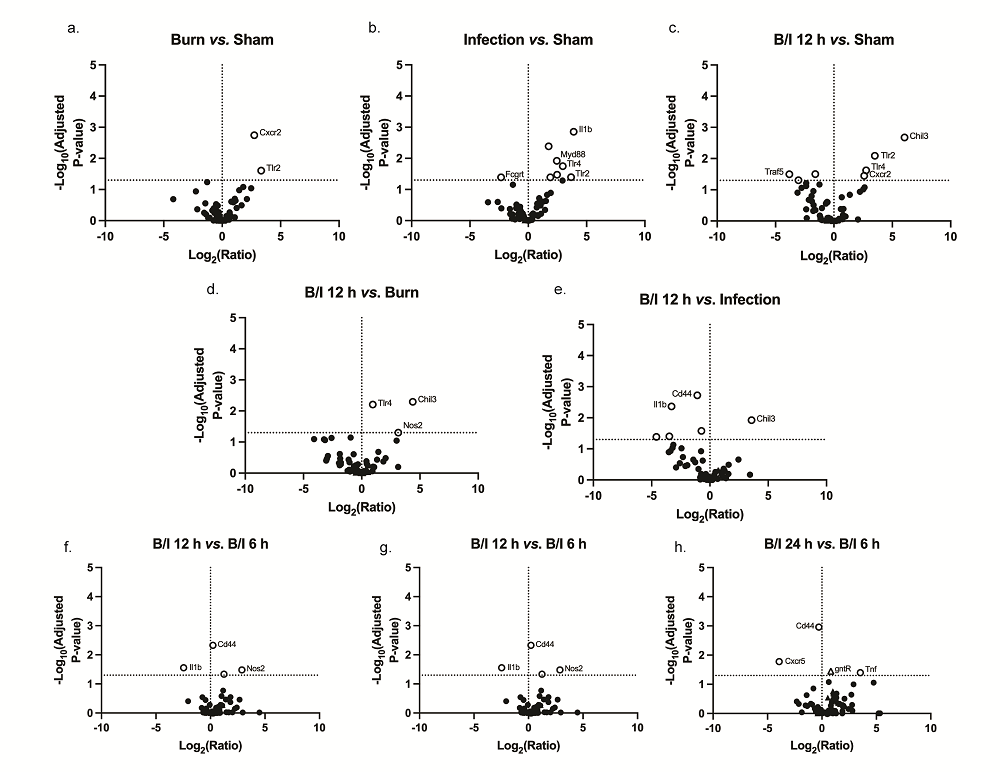

Supplement: Figure S1 — Change in gene expression in the blood in Burn alone, Infection alone, or B/I conditions compared to Sham. Experiment completed and figures prepared as described in Fig. 1. There were minimal gene changes in the blood, but each condition affected the blood differently (a-c). The B/I condition was more similar to Burn alone and Infection alone, with minimal gene changes in the B/I conditions (d, e). There was no discernible pattern of expression over time in the blood; however, by 24 h, a P. aeruginosa gene, gntR, a transcriptional regulator involved in glucose metabolism, was significantly upregulated (f-h). [file mbio.02454-23-s0001.tif]

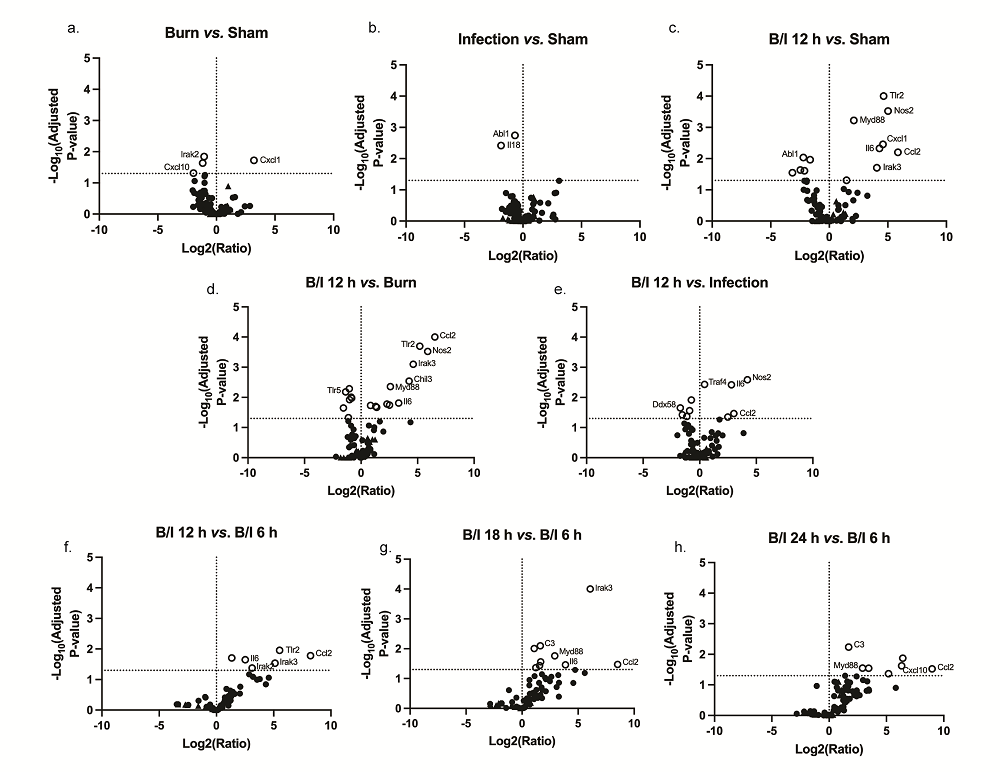

Supplement: Figure S2 — Change in gene expression in the liver in Burn alone, Infection alone, or B/I conditions compared to Sham. Experiment completed and figures prepared as described in Fig. 1. There were more genes affected in the B/I condition in both up- and downregulated genes compared to either Burn alone or Infection alone (a-c). In reference to both Burn alone and Infection alone, the B/I condition caused more upregulation of pro-inflammatory mediators (d, e). Host gene expression, but not P. aeruginosa, increased in ratio over time but not in the number of significantly expressed genes (f-h). [file mbio.02454-23-s0002.tif]

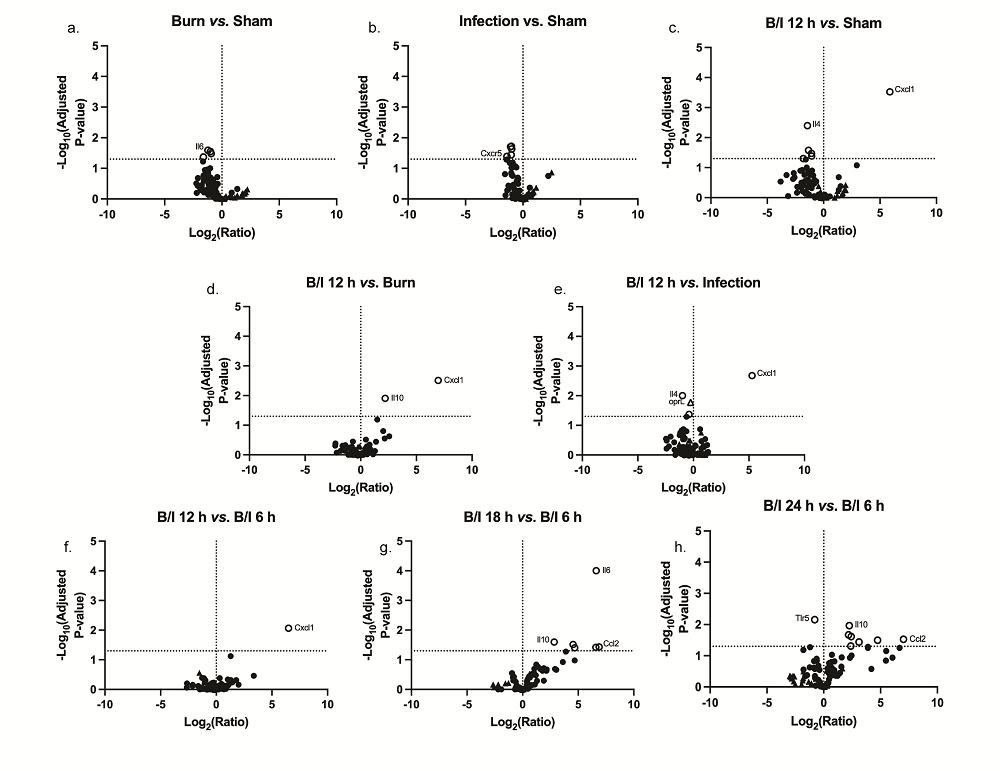

Supplement: Figure S3 — Change in gene expression in the spleen in Burn alone, Infection alone, or B/I conditions compared to Sham. Experiment completed and figures prepared as described in Fig. 1. There is only one upregulated gene in any of the conditions, Cxcl1 in the B/I condition. All other conditions caused a downregulation of genes (a-c). There was an increase of Cxcl1 over both the Burn alone and Infection alone, however there was a downregulation of oprL compared to Infection alone. Il10 was increased in expression when compared to the Burn alone condition (d, e). Upregulated host genes increased in number over time, but there were no pattern in ratio changes. Il10 expression was upregulated and maintained through 24 h post-B/I (f-h). [file mbio.02454-23-s0003.tif]

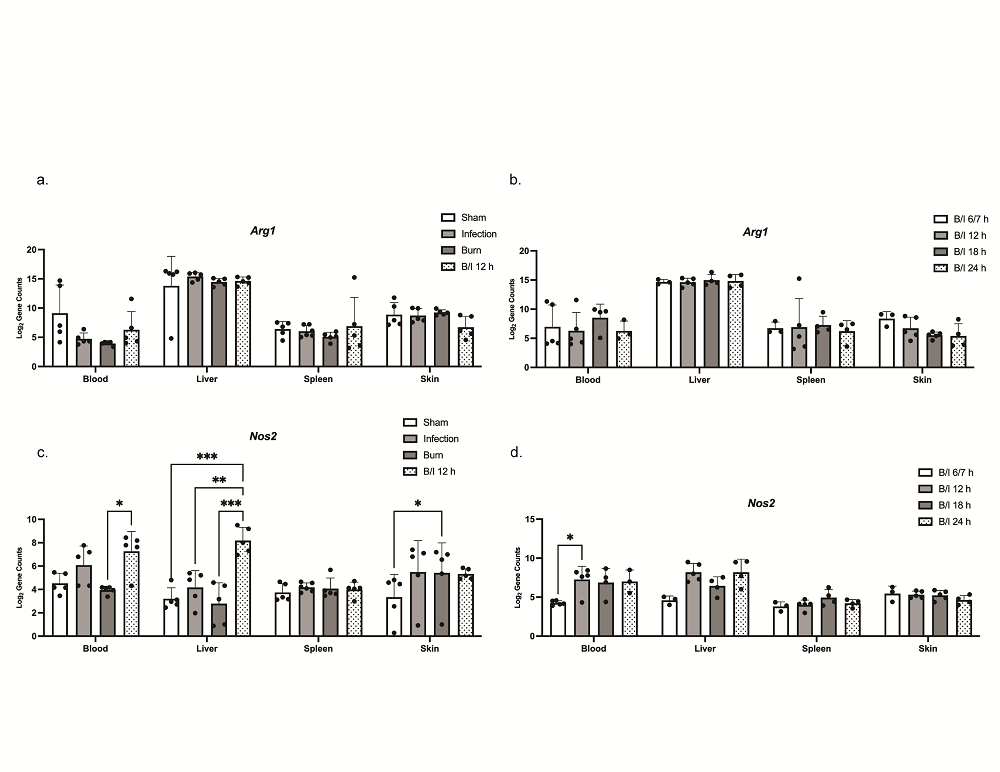

Supplement: Figure S4 — Changes in gene expression of Arg1 and Nos2 in each condition and over time in the B/I condition. The bars represent the mean of the log2 of Arg1 and Nos2 gene transcript counts in each tissue and each dot represents an individual mouse. Error bars indicate standard deviation. Significance was determined using an REML mixed model with multiple comparisons (16). There was no significant increased expression of Arg1 in any treatment condition (a) or any significant changes in expression over time in the B/I condition (b) in any tissues. Nos2 was significantly expressed in the liver and blood in the B/I condition (c) but not in the skin. Its expression increased from 6-12 h in the blood (d). [file mbio.02454-23-s0004.tif]

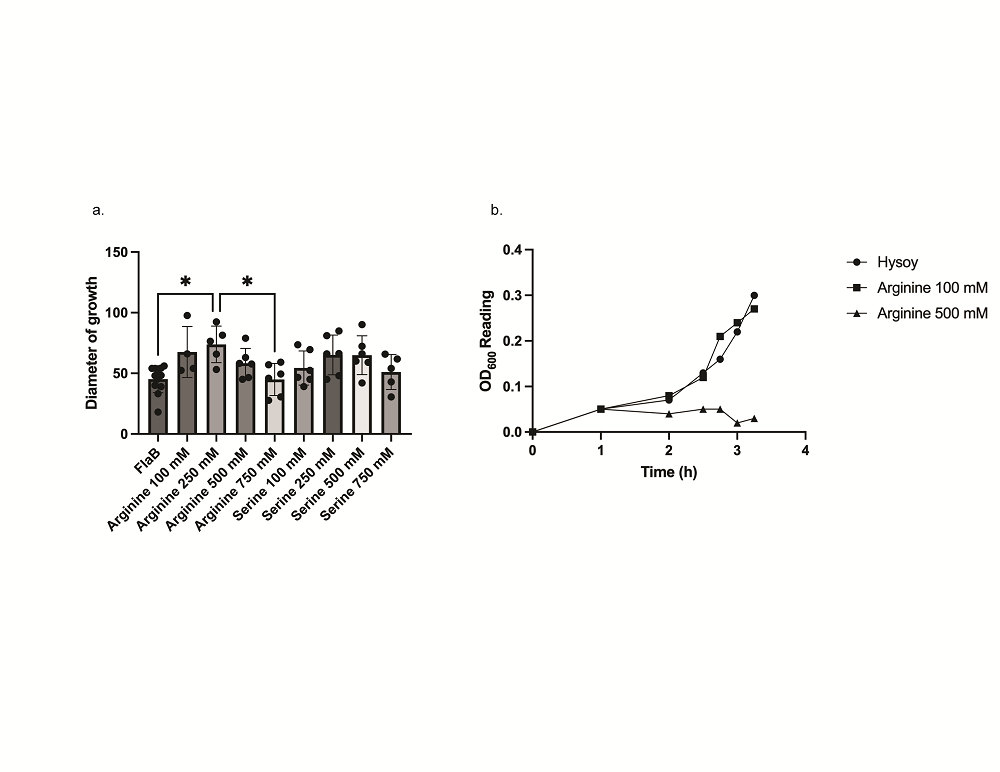

Supplement: Figure S5 — Arginine inhibits motility and growth of P. aeruginosa. (a) P. aeruginosa was grown in tryptic soy agar (TSA) wells supplemented with rabbit sera with FlaB antibodies (FlaB), or increasing doses (100-750 mM) of arginine or serine diluted in normal rabbit serum. P. aeruginosa had high motility at 250 mM which decreased in a dose-dependent manner at 750 mM. Serine did not have this effect. (b) P. aeruginosa grown in the bacterial culture medium, Hy-Soy broth, supplemented with 100 mM or 500 mM of arginine. Spectrophotometer readings were taken at OD600 to measure Pseudomonas growth. The lower dose of arginine allowed growth at a rate comparable to that in Hy-Soy broth; however, the higher dose inhibited growth. One-way ANOVA and Tukey's test with multiple comparisons. *, P, < 0.05; **, P, < 0.01; ***, P, < 0.001. [file mbio.02454-23-s0005.tif]
