# Supplementary material for: A combination of burn wound injury and Pseudomonas infection elicits unique gene expression that enhances bacterial pathogenicity
Source: mBio. 2023 Nov 6;14(6):e02454-23. doi: 10.1128/mbio.02454-23 (PMC10746159; doi:10.1128/mbio.02454-23)
Supplement: Table S1 — M. musculus genes included in NanoString panel. [file mbio.02454-23-s0006.pdf]

Official Symbol	Official Full Name
Abl1	c-abl oncogene 1, non-receptor tyrosine kinase
Bcl2	B cell leukemia/lymphoma 2
C1qa	complement component 1, q subcomponent, alpha polypeptide
C3	complement component 3
C8a	complement component 8, alpha polypeptide
C9	complement component 9
Casp1	caspase 1
Casp3	caspase 3
Casp8	caspase 8
Ccl2	chemokine (C-C motif) ligand 2
Ccl20	chemokine (C-C motif) ligand 20
Cd40	CD40 antigen
Cd44	CD44 antigen
Cxcr1	chemokine (C-X-C motif) receptor 1
Cxcr2	chemokine (C-X-C motif) receptor 2
Cxcr5	chemokine (C-X-C motif) receptor 5
Ddx58	DEAD (Asp-Glu-Ala-Asp) box polypeptide 58
Fasl	Fas ligand (TNF superfamily, member 6)
Fcer1a	Fc receptor, IgE, high affinity I, alpha polypeptide
Fcer1g	Fc receptor, IgE, high affinity I, gamma polypeptide
Fcgr1	Fc receptor, IgG, high affinity I
Fcgr2b	Fc receptor, IgG, low affinity IIb
Fcgr3	Fc receptor, IgG, low affinity III
Fcgr4	Fc receptor, IgG, low affinity IV
Fcgrt	Fc receptor, IgG, alpha chain transporter
Ifng	interferon gamma
Il10	interleukin 10
Il18	interleukin 18
Il1a	interleukin 1 alpha
Il1b	interleukin 1 beta
Il4	interleukin 4
Il6	interleukin 6
Irak1	interleukin-1 receptor-associated kinase 1
Irak2	interleukin-1 receptor-associated kinase 2
Irak3	interleukin-1 receptor-associated kinase 3
Irak4	interleukin-1 receptor-associated kinase 4
Myd88	myeloid differentiation primary response gene 88
Nod2	nucleotide-binding oligomerization domain containing 2
Pecam1	platelet/endothelial cell adhesion molecule 1
Rag1	recombination activating gene 1
Rag2	recombination activating gene 2
Tgfb1	transforming growth factor, beta 1
Tlr2	toll-like receptor 2
Tlr4	toll-like receptor 4
Tlr5	toll-like receptor 5
Tlr9	toll-like receptor 9
Tnf	tumor necrosis factor
Traf1	TNF receptor-associated factor 1
Traf2	TNF receptor-associated factor 2
Traf3	TNF receptor-associated factor 3
Traf4	TNF receptor associated factor 4
Traf5	TNF receptor-associated factor 5
Traf6	TNF receptor-associated factor 6
Ptgs2	prostaglandin-endoperoxide synthase 2
Cxcl1	chemokine (C-X-C motif) ligand 1
Nos2	nitric oxide synthase 2, inducible
Cxcl10	chemokine (C-X-C motif) ligand 10
Pparg	peroxisome proliferator activated receptor gamma
Igf2r	insulin-like growth factor 2 receptor
Il13	interleukin 13
Arg1	arginase, liver
Retnla	resistin like alpha
Chil3	chitinase-like 3
