# Supplementary material for: A combination of burn wound injury and Pseudomonas infection elicits unique gene expression that enhances bacterial pathogenicity
Source: mBio. 2023 Nov 6;14(6):e02454-23. doi: 10.1128/mbio.02454-23 (PMC10746159; doi:10.1128/mbio.02454-23)
Supplement: Table S2 — P. aeruginosa genes included in NanoString panel. [file mbio.02454-23-s0007.pdf]

Official Symbol	Official Full Name
exoT	putative exoenzyme T
lasI	N-(3-oxododecanoyl)-L-homoserine lactone synthase @ N-acyl-L-homoserine lactone synthase, LuxI family (EC 2.3.1.184)
pcrV	Tol-Pal system peptidoglycan-associated lipoprotein PAL
phoP	Type III secretion cytoplasmic LcrG inhibitor (LcrV,secretion and targeting control protein, V antigen)
fliH	Opacity protein and related surface antigens
pilQ	Flagellar basal body-associated protein FliL
tse3	GDP-mannose 6-dehydrogenase (EC 1.1.1.132)
plcH	Type IV pilus biogenesis protein PilQ
plCN	Bacterial lysozyme Tse3, effector of type VI secretion system
eptA	Phospholipase C (EC 3.1.4.3) => hemolytic PlcH
pchC	Phospholipase C (EC 3.1.4.3) => non-hemolytic PlcN
feoB	Lipid A phosphoethanolamine transferase, putative
cupB5	Pyochelin biosynthetic protein PchC, predicted thioesterase @ Thioesterase in siderophore biosynthesis gene cluster
pvdL	Ferrous iron transporter FeoB
phzS	Large exoproteins involved in heme utilization or adhesion
ftsX	Pyoverdine chromophore precursor synthetase PvdL @ Siderophore biosynthesis non-ribosomal peptide synthetase modules
tolC	FAD-dependent monooxygenase PhzS
rhaS	Cell-division-associated, ABC-transporter-like signaling protein FtsX
pvdE	Type I secretion outer membrane protein, TolC family
pilQ	T6SS PAAR-repeat protein / RhaS protein
BNR	PvdE, pyoverdine ABC export system, fused ATPase and permease components
pilC	Type IV pilus biogenesis protein PilQ
t1SS	FIG002465: BNR repeat protein
pvdL	Type IV fimbrial assembly protein PilC
pcaH	T1SS secreted agglutinin RTX
cobH	Pyoverdine sidechain non-ribosomal peptide synthetase PvdI @ Siderophore biosynthesis non-ribosomal peptide synthetase modules
gntR	Protocatechuate 3,4-dioxygenase beta chain (EC 1.13.11.3)
lysR	Precorrin-8X methylmutase (EC 5.4.99.61) biosynthetic pathway to cobalamin (vitamin B12) in aerobic bacteria
pmrA	Transcriptional regulator, GntR family
pagL	Transcriptional regulator, LysR family
lpxC	Two-component system response regulator QseB
fabZ	Lipid A 3-O-deacylase
waaA	UDP-3-O-[3-hydroxymyristoyl] N-acetylglucosamine deacetylase (EC 3.5.1.108)
ftsH	3-hydroxyacyl-[acyl-carrier-protein] dehydratase, FabZ form (EC 4.2.1.59)
pagP	3-deoxy-D-manno-octulosonic acid transferase (EC 2.4.99.12)(EC 2.4.99.13)
FtsH	Cell division-associated, ATP-dependent zinc metalloprotease FtsH
PagP	hypothetical protein (PagP)
