# Supplementary material for: A combination of burn wound injury and Pseudomonas infection elicits unique gene expression that enhances bacterial pathogenicity
Source: mBio. 2023 Nov 6;14(6):e02454-23. doi: 10.1128/mbio.02454-23 (PMC10746159; doi:10.1128/mbio.02454-23)
Supplement: Table S3 — M. musculus and P. aeruginosa housekeeping genes included in NanoString panel. P. aeruginosa genes are underlined. [file mbio.02454-23-s0008.pdf]

Internal Reference Genes	Official Full Name
Gapdh	glyceraldehyde-3-phosphate dehydrogenase
Polr1b	polymerase (RNA) I polypeptide B
Rpl19	ribosomal protein L19
Tbp	TATA box binding protein
oprL	Tol-Pal system peptidoglycan-associated lipoprotein PAL
algD	GDP-mannose 6-dehydrogenase (EC 1.1.1.132)
