# Supplementary material for: A combination of burn wound injury and Pseudomonas infection elicits unique gene expression that enhances bacterial pathogenicity
Source: mBio. 2023 Nov 6;14(6):e02454-23. doi: 10.1128/mbio.02454-23 (PMC10746159; doi:10.1128/mbio.02454-23)
Supplement: Table S4 — Number of mice sampled in each reference condition and tissue. [file mbio.02454-23-s0009.pdf]

	Blood	Liver	Spleen	Skin
Sham	5	5	5	5
Burn	5	5	5	5
Infection	5	5	6	5
