# Supplementary material for: A combination of burn wound injury and Pseudomonas infection elicits unique gene expression that enhances bacterial pathogenicity
Source: mBio. 2023 Nov 6;14(6):e02454-23. doi: 10.1128/mbio.02454-23 (PMC10746159; doi:10.1128/mbio.02454-23)
Supplement: Table S5 — Number of mice sampled in the B/I condition in each tissue per timepoint. [file mbio.02454-23-s0010.pdf]

	0 Hr	2 Hr	6 Hr	7 Hr	12 Hr	18 Hr	20 Hr	22 Hr	24 Hr
Blood	2	2	3	2	5	4	2	2	3
Liver	0	0	3	0	5	4	2	2	4
Spleen	0	0	3	0	5	4	2	1	4
Skin	0	0	1	3	5	5	3	3	4
